# Heartfulness Meditation: A Yogic and Neuroscientific Perspective

**DOI:** 10.3389/fpsyg.2022.806131

**Published:** 2022-05-10

**Authors:** Annelies van’t Westeinde, Kamlesh D. Patel

**Affiliations:** ^1^Pediatric Endocrinology Unit (QB83), Department of Women’s and Children’s Health, Karolinska Institute, Karolinska University Hospital, Stockholm, Sweden; ^2^Heartfulness Institute, Kanha Shanti Vanam, Hyderabad, India

**Keywords:** heartfulness, meditation, consciousness, brain, well-being, Transmission

## Abstract

Today, as research into the contemplative sciences is being widely referenced, the research community would benefit from an understanding of the Heartfulness method of meditation. Heartfulness offers an in-depth experiential practice focused on the evolution of human consciousness using the ancient technique of *Pranahuti* (yogic Transmission) during Meditation, in combination with the more active mental practice of “Cleaning.” Both are enabled by initiation into the Heartfulness practices. These unique features distinguish Heartfulness from other paths that have been described in the scientific literature thus far. In this introductory paper, we present the Heartfulness practices, the philosophy upon which the practices are based, and we reflect on the putative mechanisms through which Heartfulness could exert its effects on the human body and mind in the light of scientific research that has been done in other meditation systems. We conclude with suggestions for future research on the Heartfulness way of meditation.

## Introduction

Heartfulness is a contemplative tradition with a global presence that enables practitioners to experience the transcendence of individual human consciousness with the use of a few simple practices (Patel and Pollock, 2018). Scientific studies of Heartfulness have started exploring the effects of these different practices on human beings (for example, Thimmapuram et al., 2017, 2019, 2020, 2021; Arya et al., 2018; Iyer and Iyer, 2019; Yadav et al., 2021). To increase our understanding of the impact of Heartfulness on a deeper level, a clear description of the different practices of this tradition is required, in addition to understanding the philosophy upon which the practices are based.

Thus far, most research has focused on the effects of contemplative practices on cognitive function, well-being, and neurophysiology, largely without considering the philosophy or tradition from which the practices originate. A true scientific understanding of spiritual practices requires acknowledging this philosophy, in addition to the neurophysiological correlates and mental states with which they might be associated. Indeed, the Heartfulness practices have been developed based on insights about human nature from the yogic research and personal experience of the spiritual teachers and their associates, without which their purpose cannot be thoroughly understood.

It is important to note that the philosophy of Heartfulness is based on the research and practical experience of yogis, not on abstract theory. Their experience relies on “direct perception” as the preferred method of insight, which in Yoga is considered to be a more accurate method of gaining knowledge than the scientific method.

To advance our scientific understanding of these spiritual practices, there is a need to integrate the descriptions of the philosophy, the experiences lived by actual practitioners through a phenomenological approach, and the mental constructs, mental states, and physiological correlates currently addressed by science. A start has been made in a recent pilot study with EEG in Heartfulness (Sankar Sylapan et al., 2020).

To achieve this, a systematic research approach is needed. Such an approach requires a detailed description of the features of the philosophy, including the goal(s) of the method, the practices and what they intend to achieve, the lived experiences of practitioners, and how these correlate with measurable variables. This is in line with the idea of taking a phenomenological approach to studying contemplative practices (Lutz et al., 2015), as opposed to trying to categorize practices with commonalities such as attention style during meditation (Lutz et al., 2008) or having the intention to transcend the mind (Travis and Shear, 2010). In most contemplative practices, it is important to consider the journey from beginner to expert: initial meditation stages might resemble states of mind that are also found in non-meditating people, for example, concentration or relaxation, while intermediate practitioners enter more stable and deep meditation states, and experts enter states in which consciousness is altered, such as when experiencing *Samadhi* (Fell et al., 2010). In Heartfulness, however, because of the effectiveness of *Pranahuti*, *Samadhi* may be experienced by beginners, so the normal sequence does not necessarily apply. In fact, this is one of the important characteristics of Heartfulness.

In the light of these insights, the present manuscript aims to: (1) describe the yogic perspective, that is, the philosophy upon which Heartfulness is based, including the expected journey of expanding consciousness, as well as the details of the different practices that facilitate this process; (2) the neuroscientific perspective, in which we summarize current knowledge on cognitive and neurophysiological correlates of other meditation practices and propose some implications for Heartfulness; (3) suggest future research on Heartfulness.

Of note, this manuscript is intended as an introduction to the Heartfulness method for the broader scientific community and to open doors for future research. It is not intended as an extensive review of various philosophies, mechanisms of action, or comparisons with other traditions. Moreover, we do not intend to develop an elaborate theoretical framework, nor do we intend to go deeper into the nature of what consciousness is itself. We have chosen, however, to provide a definition of consciousness (Patel, informal): “Even though the ancient texts say that it cannot really be defined, I have tried to define consciousness in the modern context. One way to look at it is that consciousness is the degree of awareness, which also means the degree of unawareness. Awareness moves from zero to infinity along a spectrum according to the types of consciousness we are experiencing.”

## The Yogic Perspective: An Introduction to Heartfulness

### Historical Roots in Raja Yoga

The Heartfulness method, also known as Sahaj Marg, which means “The Natural Path,” stems from the tradition of Raja Yoga. References are found in Patanjali’s Yoga Sutras, which have been simplified and optimized into a set of daily practices by the founder of Sahaj Marg, Ram Chandra, also known as Babuji, in the early 1940s (Chandra, 1989). Patanjali describes eight limbs in his Yoga Sutras, which together form Ashtanga Yoga—the eight-limbed Yoga system (Bryant, 2009).

The first four steps—*Yama* (good conduct and removal of unwanted tendencies) and *Niyama* (cultivation of nobility of character, virtues, positive duties, and inner observances), *Asana* (refining the physical body by aligning the posture) and *Pranayama* (refining the energy body by aligning the energy flow and breath)—are outer practices traditionally considered to be preparatory steps for meditation.

Active regulation of the mind begins at the fifth step of *Pratyahara*, where the practitioner turns the senses inwards to prepare for meditation. A supposition follows (the sixth step of *Dharana*), initiating the flow of intention and direction of thought toward the object of meditation. *Dharana* leads to concentration, enabling the practitioner to have their attention and focus on an object of choice, which in turn deepens into meditation (*Dhyana*), described as the uninterrupted focus and state of absorption in the object of meditation. Finally, the state of *Samadhi* arises when a state of oneness is experienced with the object of meditation. In common understanding, *Samadhi* is considered to be an altered state of consciousness associated with a deep internal Self. While it does alter our consciousness, it is the most natural state of consciousness we can experience. It is more refined, and an evolutionary shift toward original consciousness.

Ultimately, the goal of Yoga, and also of several other contemplative traditions, is that of “union with Universal Consciousness.” This is a state of oneness achieved through purifying and refining individual consciousness so that it merges with the Universal Consciousness by means of the eight steps of Patanjali.

### The Goal of Heartfulness

Heartfulness incorporates all eight limbs of Ashtanga Yoga, but unlike many Yoga traditions, it starts with the contemplative practices first—the sixth, seventh, and eighth limbs, *Dharana, Dhyana*, and *Samadhi*. The rationale behind this is that once consciousness is purified and refined, the other five limbs can be taken up and assimilated more easily and effectively.

Heartfulness starts with meditation supported by yogic Transmission. Transmission has been defined as “divine energy from the Source, that may be used for the transformation of the human being” (Patel and Pollock, 2018). Here we are not speaking of physical transformation, as our bodies are limited by our genetic makeup. On the mental level, there are fewer limitations, but it is on the spiritual level that there is infinite scope for growth. Transmission is the nourishment that allows such unlimited growth. Transmission was re-discovered by Babuji’s teacher, Shri Ram Chandra, who was known as Lalaji. Transmission makes meditation dynamic, bringing awareness, and expands consciousness during meditation.

There are three main techniques in the Heartfulness tradition: (1) Meditation, (2) Cleaning, and (3) Prayer. These practices have the purpose of purifying and expanding the field of consciousness, until the Goal is reached, oneness with “Universal Consciousness,” known in many traditions as God, the Source, or the Ultimate. The combination of Meditation with Transmission and Cleaning aims at the purification of the “field of consciousness” by the removal of samskaras during Cleaning, thus leading to a natural expansion of consciousness as a result of Meditation (Chandra, 1989).

Note that the term “consciousness” in Heartfulness is not understood in the way that consciousness is understood by contemporary neuroscience. It is more aligned with the descriptions of Swami Vivekananda (1947), as well as the idea of a spectrum of consciousness as described by Ken Wilber (2012). The expansion of consciousness also follows an evolutionary trajectory through the chakra points (energy vortices) in the regions of the human system associated with the heart and mind. Such points are suggested to exist on a subtle energetic level, currently not well-researched by modern science, but described in a very scientific way and in great detail by Babuji.

### Subtle Energy System

The subtle energetic system of the human being is known as spiritual anatomy in Heartfulness, and is described as containing certain subtle bodies. The refinement of these subtle bodies through practice is said to support the expansion of consciousness. The idea of the human energy system with subtle bodies has been given huge importance in Eastern religions and philosophies such as Hinduism, Jainism, Buddhism, and Taoism. Yogic texts describe them as the energy field with specific focal points, known as the chakras or energy vortices (Radhakrishnan, 1994).

Early descriptions of the subtle bodies are found in the Upanishadic texts, such as the Brihadaranyaka Upanishad, Katha Upanishad, and the Taittiriya Upanishad (Radhakrishnan, 1994). For example, five energetic sheaths known as *koshas* are described in these texts. In addition, four main subtle bodies are described, namely *Chit* (consciousness), *Manas* (mind), *Buddhi* (intellect), and *Ahankar* (ego). In Heartfulness, it is understood that the functions of mind, ego, and intellect exist within the canvas of consciousness, and their refinement leads to the expansion of consciousness. Refinement of these four subtle bodies is said to happen with consistent Heartfulness practice. Specific and gradual changes in the subtle bodies influence the transcending states of consciousness experienced by the Heartfulness practitioner. The ego is our sense of identity, and it evolves through practice to identify with the Goal, the Universal Consciousness, resulting in a loving, humble, and inclusive consciousness. The intellect is our discerning faculty, and it evolves toward wisdom and clarity. The mind expands from thinking to encompass feeling and intuition, resulting in broader and less biased perspectives. As these three subtle bodies are refined, consciousness refines, and expands.

### Samskaras

Importantly, the philosophy proposes that consciousness is disturbed by energetic turbulence, much like the ocean is turbulent during a storm, due to the many impressions that are formed daily. When these impressions are not removed, they lodge in the human system creating heaviness, and eventually gain density and solidity, thereby creating heaviness in the energy system, where the impressions lodge. These impressions are known in the yogic literature as samskaras, which we will call impressions in this manuscript. The presence of impressions creates a vicious cycle; when they are triggered, they steer our behavior of approach and avoidance, and repeated experiences of the same emotions and/or events deepen the impressions. The practice of Heartfulness Cleaning is designed to remove the impressions from the system. In other words, the energetic blockages that form in the chakras are cleared, allowing for the subsequent removal of the emotional reactivity and repetitive behavioral response patterns that were associated with them.

### The Relevance of the Chakras

Although only seven chakras are described in traditional yogic literature, many more have been identified through the experience of advanced yogis, and 16 chakra points are referred to in the Heartfulness philosophy. In particular, 13 of them are associated with the human system from the heart up to the back of the head (Chandra, 1989).

The heart chakra, or *Anahata Chakra*, is the central focus of Heartfulness Meditation. It is described as a vast region which itself contains 5 sub-chakras, which we will here call chakras. Heartfulness starts with these five chakras in the region of the heart. They are associated with the five elements—earth, air, fire, water, and ether. The first chakra of the heart, found on the lower left side of the chest, has the earth element dominant. Practitioners observe that most of their impressions lodge here. Those impressions are typically associated with likes and dislikes, the pushes and pulls of desires, the effect of day-to-day worldly worries, and heavier emotions like guilt and shame. When this point is purified through the Cleaning practice, the heaviness is cleared, and when consciousness at that point is expanded through Meditation, a natural sense of contentment develops. Sensitive practitioners of Heartfulness report that they are able to relate the feeling of contentment to the specific location in the chest where this chakra is found.

In a similar way, the Cleaning process is observed to progressively purify the chakras. Importantly, through the combination of Cleaning and Meditation, as the chakras are purified, consciousness can “expand” from one chakra to the next (Chandra, 1989). After the contentment of chakra 1^1^, practitioners describe a feeling of calm at chakra 2, which is followed by the experience of love and compassion at chakra 3, then a sense of courage and confidence at chakra 4, and clarity at chakra 5. Chakra 5 is known in traditional Yoga as the throat chakra.

The above-described sequence has been repeatedly documented in detail by Heartfulness practitioners. After chakra 5, practitioners then enter the higher chakras associated with the region of the Mind. The journey through all the chakras ends at chakra 13^2^, at the back of the head, which is associated with the region of the Source itself.

Those who reach this state cannot describe the condition in words, as it is beyond qualities. They do, however, explain that it affords them 360-degree consciousness, stability and centeredness, and is the ultimate state of consciousness resembling the state before the creation of the universe, namely, the state of absolute stillness (Chandra, 1989). This state is experienced as being beyond Self-Realization and the transcendence of duality, which are known goals of other systems. The goal of Heartfulness is not specifically to reach a state of equanimity and improved well-being, although these do emerge as a result of the practices.

## The Yogic Perspective: An Introduction to Heartfulness Practices

Practitioners of Heartfulness use three main techniques to support this development of consciousness, which are Meditation, Cleaning, and Prayer.

### Heartfulness Meditation

Heartfulness Meditation is designed to help practitioners regulate and relax the mind and expand consciousness, eventually leading to a permanently awakened state, not only during meditation but at all other times. Two distinguishing features of this heart-based practice are the passive attitude of the practitioner and the use of yogic Transmission. Simple intention-setting is used only at the start, to suppose the presence of the light within the heart, also referred to as the Source or “inner Self.” The “light” in the heart is the object of meditation. It is not intended to be a visualization of light, but rather the subtlest suggestion of lightness and purity, closely associated with the quality of nothingness that is described by the Heartfulness philosophy as the Original Source.

After the initial intention-setting, the practitioner goes beyond the thought level to *experience* the object of meditation. In other words, the practitioner assumes a receptive-attentive role to allow yogic Transmission to take their awareness to deeper levels or dimensions of Self, leading in a natural manner to expansion of consciousness. The use of Transmission is the most distinguishing feature of Heartfulness and sets it apart from all other paths.

It is difficult to understand Transmission in comparative terminology, but the spiritual teachers who discovered it, and who utilize it, describe it as having zero energy and infinite speed, that is, it is instantaneous in its effect. As this concept goes beyond the realm of physics, Transmission cannot be measured or described in such terms. However, its effects can be felt, and practitioners sometimes describe a feeling of deep inner spaciousness. Transmission is said to help practitioners enter a state of meditation more easily, and facilitate the state of *Samadhi*, oneness with the Origin or Source.

The premise is that the practitioner aims to attain a state akin to the Source by setting their intention, while the Transmission emanating from the Source automatically brings them closer to the aforementioned state of consciousness. Heartfulness practitioners experience that meditation with yogic Transmission facilitates heightened awareness and glimpses of very evolved states of consciousness, including utter peace and stillness.

Heartfulness Meditation may be practiced on one’s own and with a certified trainer. When meditating with a trainer, Transmission is triggered by the trainer and is observed to flow from the Source *via* the heart of the trainer to the heart of the practitioner (Patel and Pollock, 2018). While ideal meditation sessions are conducted face to face, there is no prescribed distance between the trainer and the practitioner. In fact, the trainer may be at the other end of the world, and yet be able to transmit instantly to the practitioner when they meditate simultaneously. Importantly, Heartfulness trainers must go through a specialized training process to be certified to transmit to practitioners.

When a practitioner meditates alone, the Transmission may also be received once they have been initiated into the method. In this case, the practitioner themselves set the intention that “Transmission is flowing” at the start of their meditation.

Practitioners are advised to meditate every day in the morning, and once or twice a week with a trainer, either individually or in a group setting.

There is no strict instruction as to the position in which to meditate, but it is advised to take a comfortable sitting position, with the back upright but not rigid, and the limbs folded inward. The preference is to sit in a meditation *Asana*, with the legs crossed, but if this is uncomfortable, sitting in a chair with ankles crossed is suggested.

Importantly, during meditation, the practitioner makes the supposition that “the source of light already present in the heart is attracting them from within” at the start of the meditation. This supposition settles the awareness in the heart. They then assume a state of mind that may best be described as receptive-attentive, with the attention remaining within the heart, observing, and allowing that which is unfolding from the heart. Only if the mind becomes too distracted by thoughts do they sometimes stop and reset themselves with the thought that they are meditating on the source of light in the heart.

Practitioners experience a shift from the thinking level to feeling, both during as well as after the meditation. The receptive-attentive nature of the meditation encourages feeling because the practitioners are instructed not to actively engage with the content that is coming up into their consciousness. Rather, they are asked to simply witness the work that is being done during meditation. This receptive witnessing differentiates Heartfulness from several meditation techniques that use cognitive control in a more active way, such as Mindfulness or Vipassana.

Because of this approach, which is designed to allow thoughts and emotions to dissipate, due to a lack of intentional cognitive control or force, Heartfulness practitioners may experience a multitude of thoughts coming up during meditation, instead of reaching a state of mental stillness or equanimity that is often sought after in other methods. Practitioners are usually instructed to let the thoughts pass without paying any attention to them, so that they will be cleared out. Inner peace and stillness, as well as a shift to feeling, may therefore be expected after the thoughts and emotions have passed.

Taking the above into consideration, Heartfulness Meditation is characterized by a unique state of attention regulation, in which the aim is to (1) Set an intention of the light (from the Source) in the heart at the beginning of the meditation practice, which enhances internalization, (2) assume a receptive-attentive role, (3) let thoughts and feelings come up and pass by, and (4) most importantly, to receive Transmission which is observed to transform individual consciousness.

Finally, practitioners are encouraged to assimilate and integrate the state of awareness they develop during meditation, and sustain that “meditative state” throughout the day. This process of maintaining and recalling the meditative state is known as “constant remembrance.” Constant remembrance is designed to help practitioners retain a natural state of lightness and peace in their day-to-day activities, without actively needing to employ cognitive control mechanisms (such as mindful awareness).

### Heartfulness Cleaning

Cleaning is an active mental exercise, which is designed to remove all the “impressions” or samskaras, acquired during the daily activities, with the help of suggestion, visualization, and positive affirmations. The removal of impressions is observed to remove the root cause of mental and emotional disturbances, as described in the sections above. That is, it is observed to remove the complexities, impurities, and heaviness in the subtle energetic system caused by experience and mental and emotional disturbance.

In psychological language, this might be described as a deconditioning process, which allows the practitioner to let go of the accumulated emotional burdens (both positive and negative) caused by events in their lives. Importantly, according to both experience and philosophy, the Cleaning practice removes the root causes of impressions from the subconscious, so that tendencies are able to “fall away.” This then paves the way for behavioral change. For the effect to be lasting, the attitude of the practitioner and their willingness to make behavioral changes is vital to prevent the impressions being recreated through habit. Like Meditation, Cleaning can be done individually and with the help of a trainer.

Practitioners are advised to do the Cleaning at the end of every day, to remove any burdens they collected during that day. The practitioner sits upright, and adds their willpower as subtly as possible to the supposition that all complexities and impurities are leaving their system as smoke or vapor from the back, from the top of the head to the tailbone. They do not think of any specific event or feeling, and do not analyze the day’s activities. Thus, the practitioner themselves may be unaware of what is being removed. If they do become aware, they learn not to engage unnecessarily with the thoughts and feelings that do come up.

The latter is important, as ruminating on any particular disturbing event may unintentionally deepen the impression, as opposed to removing it. After the removal phase of the Cleaning process is completed, the practitioner then supposes that a current of purity is coming from the Source and entering the heart from the front. This current flows throughout their system, carrying away any remaining complexities and impurities. They are then advised to finish with the conviction that the Cleaning has been completed effectively (Patel and Pollock, 2018).

In addition, a practitioner can have one-to-one sessions with a certified Heartfulness trainer, during which the trainer endeavors to remove those older deeper impressions from the practitioner’s subconscious that are ready to be released (as described above). The practitioner relaxes and meditates while the trainer removes the impressions. Although this may seem improbable to modern science, its effects can nonetheless be experienced and studied.

### Heartfulness Prayer

The Heartfulness Prayer is done just before going to bed, during which the practitioner recalls the main purpose of human existence and strengthens their connection with the Source. Prayer is seen to support the experience of Meditation and the effectiveness of the Cleaning (Patel and Pollock, 2018). Practitioners are advised to repeat the words of the prayer silently and internally, and then meditate for 10 minutes on their meaning, getting lost in the words rather than trying to understand them intellectually.

The words of the prayer are:

O Master! Thou art the real goal of human life.

We are yet but slaves of wishes putting bar to our advancement.

Thou art the only God and Power to bring us up to that stage.

This short practice is meant to help the practitioner connect with the Source before sleeping, and to create a vacuumized state that opens the heart to greater and greater receptivity and empathy.

## The Neuroscientific Perspective: A Brief Overview of Meditation Research and Its Implications for Heartfulness

Scientific research can shed light on the effects of the Heartfulness practices on human physiology and psychology, gaining insight into novel dimensions of human existence that have thus far remained unmapped and little understood.

Contemporary neuroscientific research on contemplative practices often assumes the premise that a change in our behavior, for example doing a specific mental exercise such as meditation, or following a specific lifestyle, or adopting an attitude such as compassion, results in a change in our mental, emotional and physiological state, and that eventually, or directly, leads to a change in consciousness.

In Heartfulness, the premise is that the discipline of daily practice, a receptive attitude, and the support of yogic Transmission and regular sessions with a trainer, help the practitioner to transform their level of consciousness. As a result of the purification and expansion of consciousness, there will be improved mental and emotional well-being, and the (neuro)physiological state of the practitioner. This process may be greatly speeded up if the practitioner is attentive to their behavior.

We will now describe the effects on human psychology and physiology that might be expected from the Heartfulness practices, based on current knowledge gained from research in other traditions. For extensive reviews of the effects of various styles of meditation and other contemplative practices please see Travis and Shear (2010), Dahl et al. (2015), Schmalzl et al. (2015), Brandmeyer et al. (2019), and Raffone et al. (2019). Here, we will briefly summarize the most consistent findings and theories that we consider to be relevant for the study of Heartfulness.

### Varying Goals of Contemplative Traditions

A challenge of understanding the mechanisms underlying different traditions is that the paths and practices serve numerous goals, which range from enhancing well-being, reducing cognitive fusion and experiential avoidance, reaching a state of mental quietude, developing expanded states of consciousness, Self-Realization, and oneness with Universal Consciousness. Improvements in well-being and cognitive and emotion regulation may naturally emerge as a by-product of meditating for a more esoteric goal.

On a mental-emotional level, most research finds that meditation improves well-being, with small to medium effects reported for mindfulness-based practices (Goyal et al., 2014). Moreover, various meditation styles have been associated with reduced loneliness, improved sleep, reduced stress, and reduced symptoms of anxiety and depression, although higher-quality studies are needed (Goyal et al., 2014; Liu et al., 2021; Sun et al., 2021; Zhang et al., 2021).

Studies conducted on Heartfulness practitioners also find positive effects, with improvement in sleep in patients with chronic insomnia (Thimmapuram et al., 2020), reduction of burnout (Thimmapuram et al., 2019), emotional wellness (Thimmapuram et al., 2017), reduced stress and improved coping and well-being in middle school students (Iyer and Iyer, 2019), reduced stress and improved mood in teenagers (Yadav et al., 2021), and positive effects within an integrative healthcare program on coping and health-related quality of life of patients with cyclic vomiting syndrome (Venkatesan et al., 2021). Most recently, virtual Heartfulness Meditation sessions with a trainer were shown to be associated with a reduction in loneliness and improvement in sleep in healthcare providers during the COVID-19 pandemic (Thimmapuram et al., 2021). Further, in a pilot study, greater well-being was found in advanced Heartfulness practitioners compared to novice practitioners (Sankar Sylapan et al., 2020).

### Meta-Awareness as a Mechanism for Improved Well-Being

Several neurophysiological theories have been developed to explain the mechanisms through which meditation and contemplative practices lead to improvements in well-being, and cognitive and emotion-related processes, regardless of whether the meditation has well-being as a final goal. One prominent theory proposes that meditation is an attention-regulation process that creates meta-awareness, thereby enhancing emotion regulation abilities, concentration, and attentional control (Travis and Shear, 2010; Dahl et al., 2015; Schmalzl et al., 2015; Brandmeyer et al., 2019; Raffone et al., 2019). This in turn may occur by reducing cognitive fusion and experiential avoidance, thus giving space to regulate emotions before practitioners become overwhelmed, and so that they can pause before reacting (Lutz et al., 2008; Dahl et al., 2015). This is related to the process of dereification, a prominent component of Buddhistic practices, in which a subject de-identifies with the experiences and ideas about themselves (Lutz et al., 2008; Dahl et al., 2015).

Heartfulness practices cultivate the quality of *Vairagya* (Patel, 2016), or “unattached attachment,” which is akin to dereification in Buddhism. They also cultivate the quality of *Viveka* (Patel, 2016), or “discernment,” which fosters meta-awareness. The Cleaning method of removing complexities and heaviness from the system on a subtle energetic level is critical to both. Practitioners observe that Cleaning leads to a reduction in emotional reactivity, and the pushes and pulls of desires. This provides a window of opportunity for behavioral change, during which they can redirect their attention and change their attitudes and habits. Thus, the process becomes bidirectional, with desires “falling away” and habits changing.

During meditation, Heartfulness practitioners learn to ignore the thoughts and feelings that surface, so they learn to disengage from distractions coming up in their consciousness. This process is not done to avoid thoughts and feelings, but rather to witness them without engaging with them. This may be considered to be an attention regulation mechanism creating meta-awareness. Moreover, as a result of the “expansion of consciousness” experienced in Heartfulness, practitioners may identify less with their thoughts and feelings the further they grow and the more they may feel connected to the inner Self.

### Present-Moment Awareness vs. Mind-Wandering

Another attribute that is hypothesized to contribute to improved well-being, and at the same time is one of the major goals of many meditation paths, is increased present-moment awareness and reduced mind-wandering (Sood and Jones, 2013). A now-famous study has linked happiness and well-being to the feeling of being in the here and now, as opposed to being lost in mind-wandering (Killingsworth and Gilbert, 2010), while excessive mind-wandering, in particular related to negative content, is commonly associated with depression (Villalobos et al., 2021). Of note, the valence of the content of mind-wandering seems to have a substantial impact, with mind-wandering about positive or engaging topics being associated with increased happiness, even when being off-task (Franklin et al., 2013). Moreover, mind-wandering is a self-referential process, which is an important cognitive skill that is needed to gain insight into oneself and understand others. In addition, it is important for creative processes (Jung et al., 2013; Beaty et al., 2014). Hence, a more nuanced view might be needed, where meditation can help balance mind-wandering and mindful-awareness (Vago and Zeidan, 2016).

Such outcomes are also noted by Heartfulness practitioners, who reach a state in which the mind gradually becomes quiet and non-reactive, as a result of Meditation, Cleaning, and the refinement of the subtle bodies. Practitioners learn to live from a center of stillness within themselves, which is associated with the present moment. From there, they may employ cognitive functions such as mind-wandering. This may be comparable to what is aimed for in other contemplative traditions, although the mechanism (practices) through which it is reached are different. At the same time, while the Heartfulness practitioner may have the feeling of “resting” in the stillness and clarity of the Center or Source, mental, emotional, and physical activities continue at the surface level. Thus, we would expect a shift in identification, as well as an expansion of the field of feeling and thinking. This, along with an expanded consciousness, may contain more information simultaneously. This idea is in line with the proposal of Raffone et al. (2019) regarding the expansion of the cognitive workspace as a result of meditation (see below).

### Neurophysiological Mechanisms Underlying the Effects of Meditation: Network Rebalancing

Neuro-imaging research has shed light on the potential mechanisms that might underlie increased meta-awareness, improved emotion regulation abilities and cognitive control, reduced mind-wandering, and increased present-moment awareness. These theories have focused both on what happens during meditation (state effects) as well as on lasting changes in brain networks (trait effects). Currently, the predominant view is that the brain is organized as a set of networks that give rise to cognition. Three major networks are: the salience network (SN), which is involved in detecting, directing attention toward, and processing internal and external salient stimuli; the default mode network (DMN), which is associated with self- and other-referential thinking; and the central executive network (CEN), which is involved in higher-order executive functions such as working memory and emotion regulation (Menon, 2011; Zhou et al., 2018).

A popular explanation of the positive effects of meditation is that it reduces the activity in the DMN, which is commonly associated with mind-wandering, in particular about negative emotions (Sood and Jones, 2013; Bartova et al., 2015; Bauer et al., 2019; Yang et al., 2019). At the same time, meditation might increase activity in the nodes of the CEN associated with attentional control, as well as with reduced activity in the limbic nodes of the SN related to responding to salient cues (Marusak et al., 2018; Sørensen et al., 2018; Bauer et al., 2019). Indeed, a recent study found a re-balancing of the three networks as a result of Vipassana meditation, wherein increased coupling between the CEN and DMN was found *during* meditation, and increased activity of the CEN and reduced coupling between the CEN and DMN *after* meditation (Bauer et al., 2019). Reduced SN activity might be interpreted as reduced responsiveness to salient cues, or greater ability to disengage from salient cues, as has been found for example in mindfulness (Sørensen et al., 2018). This could in turn lead to less unnecessary rumination involving the DMN, which frees energy resources for higher-order cognitive functions (Raffone et al., 2019).

These theories have interesting implications for Heartfulness practices, which we expect to be associated with changes in these networks. The philosophy states that Heartfulness works at the level of the subtle body, which then affects every atom of the physical body, including the whole of the nervous system. The characteristic attitude during Meditation of inward attention on the heart, and the effect of Transmission, may have implications for DMN network activity. While we may expect a strong CEN component during Heartfulness Meditation, we also expect to see a bottom-up change in activity, although predictions are hard to make and exploratory imaging research is needed. Further, as the Cleaning practice aims to remove the root cause of emotional reactivity, it may be associated with reduced SN activity that would otherwise trigger DMN activity, in turn allowing space for the CEN to focus (Sørensen et al., 2018; Bauer et al., 2019).

The attitude of the practitioner is critical here, as they need to actively change their habits for the effects to be lasting. Otherwise, the impressions may re-form if the practitioner’s habits and tendencies do not change. At the same time, as Cleaning employs willpower, it may be associated with CEN components during the exercise itself, albeit without trying to intentionally bring any cognitive and emotional content into awareness. The observations of practitioners, and the philosophy based on the research of the Heartfulness teachers indicate that the Cleaning practice at the end of the day reduces the formation of impressions from emotional responses. Hence, we may expect to find a change in the coupling between the prefrontal cortex, amygdala, and hippocampus as a result of Cleaning (Anderson and Floresco, 2021; Roesler et al., 2021; Szadzinska et al., 2021). A sound theoretical cognitive framework, incorporating these perspectives on expanding consciousness, is needed to fully appreciate the interplay of Heartfulness Meditation, Cleaning, and expansion of consciousness.

### Neurophysiological Mechanisms Underlying the Effects of Meditation at a Neural Level: Freed Energy Resources

As the mind becomes quiet and non-reactive as a result of meditation, energy is freed for improved working memory and focus (Lutz et al., 2019; Raffone et al., 2019). For example, improved concentration and attentional stability are found after Vipassana training (Zanesco et al., 2013). In their brain theory of meditation, Raffone et al. (2019) propose that training the mind through meditation can expand and optimize the cognitive workspace by efficient allocation of energy resources to relevant tasks. This idea is in line with other recently proposed theories related to the minimization of free energy, in which the state of mental quietude reached through meditation entails that the brain learns to optimize energy usage by minimizing prediction errors (Lutz et al., 2019). Importantly however, the mechanism through which this state of mind is reached may differ with the method of meditation used.

For example, Raffone et al. (2019) propose that focused attention meditation employs top-down regulation of network activity, while open-monitoring meditation employs a change in sensory gating. In addition, this learning process in attention-based meditation styles depends on consciously bringing into awareness the cognitive content, such as thoughts and emotions, that otherwise largely happens unconsciously or in a state of cognitive fusion and experiential avoidance. This is probably why increased activity in the DMN is found, for example, during Vipassana meditation, which is characterized by a strong self-referential process of labeling cognitive content (Travis and Shear, 2010; Bauer et al., 2019).

Importantly, the purpose of Heartfulness Meditation is to eventually transcend these mental processes and focus on the higher goal of the Source. In this regard, a strong referential process toward cognitive content is not encouraged during the meditation itself. You may therefore think that neither focused attention nor the bringing into consciousness of cognitive content are given importance in Heartfulness, but this would not be true. In fact, an emphasis on the first two limbs of Ashtanga Yoga, *Yama* and *Niyama*, is critical to the Heartfulness methodology. All practitioners are requested to write a journal to practice *swadhyaya*, or self-study, and to do other practices that focus on continuous improvement in character. As well as the core practices mentioned—Meditation, Cleaning, and Prayer—even greater importance is given to character and lifestyle, and a set of Ten Maxims is given for guidance. Thus, an expanded cognitive workspace, mental quietude, decreased sensitization, and minimized prediction errors do happen along the way, as a result of the combined Heartfulness practices. This is of interest to investigate in future research.

### Neural Correlates of the Expanding Consciousness in Different Paths

What development of consciousness really entails from a scientific perspective remains the million-dollar question. Neural signatures, both during and after meditation, may reflect a change in consciousness, in particular in the paths that are aiming to transcend duality, and these signatures or correlates are expected to differ according to the practices done. For example, in terms of state effects during meditation, the difference in the extent of attentional control used during meditation between focused attention (FA), open monitoring (OM), and automatic self-transcending (AST) has been proposed to be reflected in enhanced beta/gamma waves for FA, theta waves for OM, and alpha waves in AST (Travis and Shear, 2010). Furthermore, one meta-analysis comparing Hindu-based and Buddhist-based functional brain activity studies found that Buddhist meditations, which traditionally have a strong mindfulness component, were more associated with activity in brain regions of the frontal lobe involved in executive function, while Hindu-based meditations, which have a focus on transcending duality, were associated with regions in a left-lateralized network, including the postcentral gyrus, superior parietal lobe, hippocampus, and right middle cingulate cortex (Tomasino et al., 2014). In other words, Hindu-inspired meditations, which focus on transcendence, are more associated with posterior regions as opposed to frontal-based Buddhist practices that focus on mindfulness. This illustrates that even if the goal is the same or similar, the road taken, the methods used, may be associated with different correlates on a mental and physiological level.

As Heartfulness evolved out of the yogic tradition, it is neither traditionally Hindu nor Buddhist, ushering in a new era of practices that integrate both. For example, mindfulness is the first step in *Pratyahara* in Ashtanga Yoga, and is very much present in the Heartfulness approach. Transcendence is equally there in the form of *Samadhi*. All eight limbs of Ashtanga Yoga are valued and integrated. So, we might expect activity in both the frontal lobes and the posterior regions of the brain. In fact, given that Heartfulness awakens all the chakras, from the base of the spine right up to the occipital prominence region of the brain, we would expect their awakening to contribute to increased functionality in a broad spectrum of the central nervous system.

In fact, categorizations like Buddhist-based and Hindu-based are oversimplifications, as a range of brainwave and/or network activity changes are observed during meditation of any style, reflecting the different mental states and stages of consciousness that practitioners move through (Fell et al., 2010; Kora et al., 2021). It is therefore important to consider the journey from beginner to expert, which may reflect a gradually changing and expanding consciousness. Fell et al. (2010) propose that for beginners, meditation states are related to cognitive processes that are also found in non-meditative exercises, that is alpha and theta. These relate to internalizing attention, relaxation, and cognitive effort, which are easiest to modulate by cognitive control (Kubota et al., 2001; Grosselin et al., 2021; McFerren et al., 2021). In intermediate practitioners, gamma waves are altered, which could be involved in reaching novel states of consciousness through learning (Axmacher et al., 2006). Finally, advanced meditation might be associated with altered states of consciousness associated with changes in alpha, theta, gamma, and even delta, thereby expanding the range of frequencies experienced, eventually even at baseline (Fell et al., 2010). This interesting theory is in line with more recent research, showing that mindfulness meditation is most commonly associated with enhanced alpha and theta (Lomas et al., 2015), while delta waves are found mostly in experienced practitioners, or during deep stages of Vipassana meditation (Kakumanu et al., 2018). However, changes in brainwaves during and after meditation are complex and might lead to a reorganization of the brain networks at baseline in terms of dynamic functional coupling, which cannot be expressed merely as a change in one or the other frequency (Bréchet et al., 2021).

Moreover, there are methodological challenges to EEG measurements during meditation, which are often confounded by muscle relaxation. These need to be addressed when studying the effects of meditation on brainwaves (Deolindo et al., 2020; Kora et al., 2021). In addition, even the specific networks involved in meditation are expected to change from beginner to expert. For example, it has been suggested that mindfulness meditation is a top-down attention regulation process for novices, associated with stronger activity in the prefrontal area of the brain, CEN associated regions, while shifting to a bottom-up approach, associated with reduced activity in limbic areas after years of practice (Chiesa et al., 2013).

On one hand, a change from beginner to expert is explicitly expected in Heartfulness, based on the journey of consciousness through the chakra points described above. Moreover, the specific goal of reaching the Source, the attitude of the practitioner, the reception of Transmission, as well as the addition of the Cleaning practice in the Heartfulness method, have implications for the networks expected to be involved and evolving in Heartfulness practitioners.

On the other hand, Transmission offers a direct link to the Source from the very first meditation sessions of a beginner, and both Gamma and Delta brainwaves have been measured in first-time meditators with Transmission, so another hypothesis would be that evolved states of consciousness will be accessible even for a beginner. This was indeed found by the pilot study on Heartfulness practitioners using EEG, where similar brain activity, including changes in Delta waves, were reported in both experienced and novice meditators while meditating with Transmission (Sankar Sylapan et al., 2020). Moreover, recent studies on Heartfulness have found a rebalancing of the Autonomic Nervous System (ANS), even in novice practitioners. Indeed, Rajlakshmi Borthakur (Patel, 2021; Borthakur et al., 2022), who has been studying the effects of Heartfulness practices using wearable devices that measure Heart Rate Variability (HRV) and Electrodermal Activity (EDA), found that in novice meditators, high frequency power was higher than controls when meditating with Transmission, while Low Frequency power was lower. These findings point at a sympatho-vagal balance that shifts to the parasympathic side (Patel, 2021; Borthakur et al., 2022). Their study also found that experienced practitioners were longer able to hold a state of calmness, meditators and trainers had increased heart rate variability, but decreased skin conductance response (SCR), and during deep meditation experienced practitioners had a decrease in their sudomotor nerve activity (SMNA).

## Future Research on Heartfulness

### The Journey of Expanding Consciousness

The more elusive parts of contemplative practices are challenging to investigate scientifically, as they deal with phenomena outside the measurable reach of contemporary science. In addition to measuring neurophysiological variables and cognitive constructs, it is therefore important to take a phenomenological approach.

#### Phenomenology of Subtle Bodies

Studying the expansion of consciousness through the chakra points is a challenge for science. Attempts have been made to relate chakra points to the existence of gap-junctions in the spinal cord (Maxwell, 2009) and other maps of the central nervous system (Loizzo, 2016). However, at the current stage, there is no convincing physiological correlate that may be used as evidence for the existence of or measurement of the chakras.

There is a major gap between what science is currently able to measure and understand, and the repeatable observations that have been described by advanced practitioners of meditation through inner research. The detailed descriptions of the subtle energy system in Heartfulness can be investigated in terms of phenomenological experiences of practitioners. This way, the subtle energetic system has been and can continue to be investigated, and we can gain insights into other realms of existence and transcendental human experiences that have largely been ignored by science so far. In addition, future research could attempt to correlate the subtle bodies, that is consciousness, mind, intellect and ego, to psychological functions, as has already been done in the yogic sciences, for example in Patanjali’s Yoga Sutras (Patel, 2019).

#### Stages of Consciousness: The Map and the Journey From Beginner to Expert

Based on the journey of expansion through the 13 chakras (see text footnote 2) from the heart upward, as described in the Heartfulness philosophy, we expect increasing changes in specific mental-emotional aspects related to the specific chakras that are traversed. These may be studied through the phenomenological descriptions of advanced practitioners of Heartfulness. Indeed, as we expect the specific chakras to be associated with certain states of mind, and improvements in certain aspects of behavior, this may also be investigated.

In addition, specific states of consciousness relating to each chakra may also be correlated with unique neural signatures. More specifically, the 13 chakras in the Heartfulness journey are from the heart to the head, with the last chakra found at the occipital prominence at the back of the head. Potentially, brain activity will change as the practitioner’s consciousness expands from one chakra to the next.

At this stage it is not possible to predict what these changes would look like. Given the importance of the last point at the back of the head, we may expect more posterior brain activity as a result of and/or during Heartfulness Meditation. Interestingly, one of the first studies on brain function in Heartfulness identified stronger Gamma oscillations specifically in the occipital lobe (Sankar Sylapan et al., 2020). However, naturally, this may also be caused by practitioners focusing on “the light in the heart.” Moreover, an obvious challenge to studying such correlates is that there is no way to scientifically measure “where” a person is situated with respect to the chakras, and it may also fluctuate from time to time, although the current spiritual Guide of Heartfulness and other experienced trainers are able to “read” this.

It may further be expected that the more expanded stages of consciousness are associated with a wider range of EEG frequencies. More advanced stages may also lead to an increasing sense of oneness and stillness. Recently, the Minimal Phenomenological Experience questionnaire (MPE-92M) was developed in order to systematically investigate the experience of oneness by practitioners (Gamma and Metzinger, 2021), and there are other methods of investigating the oneness experience (Van Lente and Hogan, 2020). A similar approach might be used to document the increased sense of oneness that results from the Heartfulness practices, as practitioners progress in the method, related to the expansion of consciousness. Identified states of consciousness, as well as changes in aspects of behavior, might then be correlated to brain function and structure. Notably, the Heartfulness practices as a whole were developed to lead to the progressive transformation of the practitioner. It is therefore important to investigate changes in the practitioner’s worldviews and lifestyle. Naturally, longitudinal studies are needed to follow individual practitioners.

### Heartfulness Meditation

Two questions of interest regarding Heartfulness Meditation are: (1) What is the effect of Heartfulness Meditation on human psychology and physiology? (2) What are the specific effects of Transmission during Heartfulness Meditation? Therefore, studies are needed in which the effects of individual meditation on psychology and neurophysiology are investigated. These would be compared with either a non-meditating control group, or another method of individual meditation, such as attention-based practices as mindfulness or Transcendental Meditation, to compare Heartfulness more directly to a meditation style that has the same goal but does not use Transmission.

In addition, Heartfulness Meditation may be done with a trainer, individually and in groups of various sizes. Meditation with a trainer in a group setting needs to be studied, which will be particularly useful for studying the effect of Transmission. To achieve this, it will be helpful to develop a standardized Heartfulness Meditation protocol, including a fixed program over a certain period. This may be used in randomized controlled trials, where novice Heartfulness practitioners are enrolled in a meditation program. Interestingly, Delorme and Brandmeyer have suggested that failure to remain equanimous with emotions and thoughts during other forms of meditation might be deleterious to the practice (Delorme and Brandmeyer, 2019). It is important to investigate this aspect in the Heartfulness practice, where practitioners learn to ignore unwanted thoughts and emotions, and there is also the support of Transmission to stay equanimous.

#### Transmission

Transmission itself cannot be measured by any means available to us, but we can observe its effects in terms of phenomenological experiences, as well as the resultant changes in well-being, cognitive function, and brain activity. As we expect that Transmission will help a practitioner reach deeper stages of meditation sooner, this may be reflected in the neural signatures. For example, we may find changes in brainwaves along a broader spectrum than usual in beginners (Sankar Sylapan et al., 2020). Furthermore, this might be transferred to a state-change with repeated practice. This can be investigated in study designs in which practitioners meditate together with a trainer, where trainers initiate the process of Transmission in one condition, and not in another.

Furthermore, one of the most interesting research possibilities of our method may be hyperscanning, in which the responses of the trainer and practitioner meditating together are measured simultaneously (Scholkmann et al., 2013). This could include the use of near-infrared spectroscopy to measure simultaneous brain activity (Crivelli and Balconi, 2017), in addition to concurrent measurement of autonomic and behavioral responses. These may be integrated to compute estimates of synchronization.

Heart resonance between trainer and practitioner may also be investigated. Finally, wearable devices for practitioners to measure heart rate variability and skin conductance response, as used by Borthakur et al. (2022), will make it possible to assess the effects of Meditation and Cleaning on day-to-day well-being (Balconi et al., 2019). The extent to which practitioners either experience or actively practice constant remembrance is also of interest to investigate, as we expect that this will greatly affect the effectiveness of the meditation practice, both in terms of well-being, as well as in speed of development of consciousness.

### Cleaning

Similar studies are needed to investigate the effects of the Cleaning when done individually and when done with a trainer, in terms of well-being, cognitive function, emotion regulation, and neurophysiological correlates. Developing a standardized protocol for research studies will be of great help here. When done with a trainer, a similar method of hyperscanning may be employed as described above. At this stage, we have not yet developed an elaborate cognitive/physiological framework to explain the effects of Cleaning, however, the purpose of removing impressions hints at some interesting hypotheses to explore.

As explained above, Cleaning removes complexities, impurities, and heaviness from the energetic system, allowing for the subsequent removal of the root cause of emotional reactivity and repetitive behavior responses associated with the removed impressions. This suggests that a relationship might exist between impressions and, for example, the formation of emotional memories and habitual or reactive behavior patterns associated with our attraction and repulsion toward things in life. This may have effects on emotional well-being and might be expected to reduce rumination regarding daily events, especially if practiced every day.

The interaction between the practice of Cleaning, observations of changes in wants and needs, and the attitude and willingness of the practitioner to change their behaviors are important to consider in future study designs. Other variables of interest to test in association with the Cleaning are stress-induced anticipatory sensitization (Turan et al., 2015), emotion regulation, and dream content. These can be tested either directly using validated experimental designs, or using participant interviews. Finally, one important aspect to consider when testing the effects of the Cleaning method is to include an active control group that is engaged in a task that requires a similar amount of concentration.

### Current Challenges

Despite the numerous research possibilities provided by this simple system of meditation, in combination with an elaborate philosophy underlying the practice, many challenges currently exist. Firstly, we are dealing with phenomena that lie outside the scope of traditional science. It is therefore important to describe accurately what we aim to study, and to differentiate between so-called “subjective” spiritual experiences (even though they may be the result of direct perception) and measurable experiences that can be assessed using experimental design. In addition, heterogeneity between practitioners provides a challenge, as practitioners differ in terms of motivation, the reasons they meditate, sensitivity, and baseline states of consciousness, before embarking on a contemplative path. Moreover, Heartfulness practitioners may be instructed about the philosophy, which could bias their self-perception.

It is also tempting to assume that meditation only has beneficial effects for the practitioner, however, anybody who has tried such practices will have experienced the challenges that exist when starting the training. Indeed, one of the studies on Heartfulness found lower well-being in novice practitioners compared to controls, albeit not significantly (Sankar Sylapan et al., 2020). Improved well-being and quality of life might be a feature that emerges with time. This is in line with the idea that Heartfulness Meditation aims to develop consciousness, which might go hand in hand with the temporary turbulence caused by the removal of impressions, and the significant changes in behavior and personality that need to take place. Indeed, meditation is a way of self-development, and the right guidance is needed to train practitioners to overcome challenges along the way.

It may be expected that many novices stop after their initial fascination has faded, as commitment to the practice and the establishment of a disciplined routine are requirements for advancement. Such attrition will have an impact on the perceived effects of the practice. An alternative explanation for the slight reduction in well-being in novice practitioners might be that people with mental health problems opt for meditation in the first place, to learn to cope with their problems. Mental health improvements are not always found in other meditation systems either, and the negative experiences associated with meditation, in particular incidences of psychosis after prolonged stays in retreat centers, require attention (Schlosser et al., 2019). These occurrences stress the importance of randomized controlled trials to assess the effects of meditation.

## Conclusion

Heartfulness is a simple meditation method that has the potential to affect human beings at many different levels of their existence. The detailed philosophy, based on practical experience, and the simple exercises offer an opportunity to study the effects of this method on human psychology and physiology in depth. In particular, the two-way interaction between the practitioner using their intention and willpower during the Meditation and Cleaning, and the effect of Transmission that is perceived by practitioners to change consciousness on a subtle level, provides for an intriguing dynamic process of change that can be investigated. In addition, insights may be gained in those realms of existence that have yet to be discovered by contemporary science. With a systematic research approach, we hope to explore the mechanisms by which these processes work in Heartfulness and contribute to the increasing understanding of the complexity of human existence.

## Data Availability Statement

The original contributions presented in the study are included in the article/supplementary material, further inquiries can be directed to the corresponding author.

## Author Contributions

AW wrote the main body of the text. KP contributed to the sections regarding the Heartfulness philosophy and contributed to and inspired the main ideas proposed in this text. Both authors contributed to the article and approved the submitted version.

## Conflict of Interest

Both authors of this manuscript were actively practicing Heartfulness meditation. KP was the president of the Heartfulness mission, and AW was an active practitioner.

## Publisher’s Note

All claims expressed in this article are solely those of the authors and do not necessarily represent those of their affiliated organizations, or those of the publisher, the editors and the reviewers. Any product that may be evaluated in this article, or claim that may be made by its manufacturer, is not guaranteed or endorsed by the publisher.

## Footnotes

1 This first chakra is in the heart region, not to be confused with *Mooladhara chakra*, which is considered the first chakra in many systems, but which resides at the base of the spine.

2 These 13 do not include the 3 chakras below the heart, *Mooladhara, Swadisthana*, and *Nabhi*.
